# Intravenous versus oral omeprazole on patients with high risk bleeding peptic ulcers: A prospective randomized clinical trial protocol

**DOI:** 10.1097/MD.0000000000025136

**Published:** 2021-04-09

**Authors:** Jing Zhang, Panya Diao, Lin Zhang

**Affiliations:** Department of Gastroenterology, Jiangjin District Central Hospital, Chongqing, China, 402260.

**Keywords:** intravenous, omeprazole, oral, peptic ulcers, proton pump inhibitor

## Abstract

**Background:**

Proton pump inhibitors (PPIs) decrease the rate of rebleeding following endoscopic hemostatic therapy in patients with bleeding peptic ulcers. This study compares the efficacy of oral omeprazole vs intravenous omeprazole in decrease of rebleeding of peptic ulcer patients.

**Method:**

The present study was authorized by the local research ethics committee of Jiangjin District Central Hospital (2020120987) and informed consent was obtained from all patients. All adult patients who were admitted to medical emergency rooms of Jiangjin District Central Hospital due to upper gastrointestinal bleeding (as evidenced by hematemesis, melena or hematochezia) were considered for inclusion in the study. Endoscopy was performed within 24 hours after admission. Patients older than 18 years with successful endoscopic therapy of high risk ulcers [defined as active bleeding (Forrest IA, IB), non-bleeding visible vessel (NBVV, Forrest IIA) or adherent clots (Forrest IIB)] were enrolled. Patients with low risk ulcers (clean base, ulcers with a simple washable clot), suspicious malignant ulcer, bleeding tendency, uremia, liver cirrhosis, Mallory Weiss tear or already on PPI as an outpatient were excluded from study. All were managed endoscopically by injecting 5–30 ml of epinephrine (diluted 1:10000) around the ulcer crater. Cavitations or flattening of bleeding vessel and disappearance of NBVV was considered as established homeostasis. A biopsy was taken from antrum for evaluating Helicobacter pylori infection. Patient with unsuccessful endoscopic therapy were not enrolled and were referred to general surgeon. Information on demography, history of previous upper gastrointestinal bleeding, NSAID or ASA ingestion, ulcer location, bleeding stigmata and blood transfusion volume at entry were recorded in all patients. In the oral omeprazole group, the patients received 40 mg omeprazole orally twice daily for 72 hours. In intravenous omeprazole group, they received omeprazole 80 mg bolus and then 8 mg/hour infusion for 48–72 hours. Then, all patients received omeprazole 20 mg orally for 30 days. On the day of discharge Helicobacter pylori infected patients received standard regimens.

**Results:**

Figure 1 showed the primary and secondary end points.

**Discussion:**

Intravenous administration of PPIs has limitations. They are expensive, require a dedicated intravenous line, need nursing supervision and hospital admission. So, it would be reasonable to prescribe oral PPIs to patients with high risk bleeding ulcers provided that it is as effective as its intravenous counterpart. Oral PPIs have a high bioavailability. Its effect initiates one hour after ingestion and the maximal plasma concentration is achieved after 2–3 hours. However, there are few studies comparing oral and intravenous PPI in decreasing risk of rebleeding in peptic ulcer patients. More high quality randomized controlled trials are still necessary.

**Registration number:**

researchregistry 6588

## 1 Introduction

Upper gastrointestinal bleeding is a common emergency with significant morbidity and mortality.^[1]^ Peptic ulcer disease is the most common cause accounting for about 50% of episodes.^[2,3]^ The estimated prevalence of peptic ulcer disease in the general population is 5–10%,^[4]^ but recent epidemiological studies have shown a decrease in the incidence, rates of hospital admissions, and mortality associated with peptic ulcer.^[5,6]^ This is most likely secondary to the introduction of new therapies and improved hygiene, which resulted in a decline in Helicobacter pylori infections.

Endoscopic therapy of high risk ulcers such as epinephrine injection reduces rebleeding, morbidity and even mortality. Therefore, it is currently recommended as the first line of hemostatic intervention for these patients. However, high risk ulcers rebleed in 14–36% of patient in spite of efficient endoscopic intervention.^[7,8]^ Gastric acid inhibits clot formation and promotes clot lyses and therefore disturbs hemostasis of ulcers in the stomach and duodenum. So reduction of gastric acid secretion could prevent ulcer rebleeding.^[9,10]^ Irreversible inhibition of the H+/K+-ATPase proton pump, which varies between proton pump inhibitors (PPIs), allows more effective, direct suppression of acid secretion.^[11,12]^ While the role of adjuvant PPIs after endoscopic therapy has been confirmed in managing bleeding peptic ulcers. Lau et al^[13]^ indicated that after endoscopic treatment of bleeding peptic ulcers, a high-dose infusion of omeprazole substantially reduces the risk of recurrent bleeding. Lin et al^[14]^ reported that oral omeprazole is effective in increasing intragastric pH and reducing rebleeding episodes in patients with bleeding peptic ulcers after successful endoscopic therapy. However, the comparable effectiveness of oral and intravenous route of administration is not well known due to the poor study design and small sample size of published articles. We perform a prospective randomized clinical trial protocol to compare the efficacy of oral omeprazole vs intravenous omeprazole in decreasing risk of re-bleeding in peptic ulcer patients.

## 2 Methods

The present study was authorized by the local research ethics committee of Jiangjin District Central Hospital (2020120987) and informed consent was obtained from all patients. Patients were enrolled in a consecutive prospective manner on a voluntary basis. The study was registered in the public trial registry (researchregistry 6588).

All adult patients who were admitted to medical emergency rooms of Jiangjin District Central Hospital due to upper gastrointestinal bleeding (as evidenced by hematemesis, melena or hematochezia) were considered for inclusion in the study. Endoscopy was performed within 24 hours after admission. Patients older than 18 years with successful endoscopic therapy of high risk ulcers [defined as active bleeding (Forrest IA, IB), non-bleeding visible vessel (NBVV, Forrest IIA) or adherent clots (Forrest IIB)] were enrolled. Patients with low risk ulcers (clean base, ulcers with a simple washable clot), suspicious malignant ulcer, bleeding tendency, uremia, liver cirrhosis, Mallory Weiss tear or already on PPI as an outpatient were excluded from study.

### 2.1 Randomization and blinding

Patients were randomly assigned on a 1:1 basis to receive either intravenous group or oral group. Randomized numbers were ranging from 0 to 99 were generated using computer software. On enrollment, a sealed envelope was selected in the pharmacy department by allocating staff who did not take part in treatment or assessment of outcomes. Patients with even numbers were allocated to the group scheduled to receive intravenous PPI, and those with odd numbers were allocated to receive oral PPI. We ensured that the patients, care providers, and outcome assessors were blinded to the group assignment during the study period. They were all unaware of the randomization given by the allocating staff. Finally, 90 patients were included in the study.

### 2.2 Treatment

All were managed endoscopically by injecting 5–30 ml of epinephrine (diluted 1:10000) around the ulcer crater. Cavitations or flattening of bleeding vessel and disappearance of NBVV was considered as established homeostasis. A biopsy was taken from antrum for evaluating Helicobacter pylori infection. Patient with unsuccessful endoscopic therapy were not enrolled and were referred to general surgeon. Information on demography, history of previous upper gastrointestinal bleeding, NSAID or ASA ingestion, ulcer location, bleeding stigmata and blood transfusion volume at entry were recorded in all patients. In the oral omeprazole group, the patients received 40 mg omeprazole orally twice daily for 72 hours. In intravenous omeprazole group, they received omeprazole 80 mg bolus and then 8 mg/hour infusion for 48–72 hours. Then, all patients received omeprazole 20 mg orally for 30 days. On the day of discharge Helicobacter pylori infected patients received standard regimens.

The patients were monitored for supine and sitting vital signs, intravenous fluid intake, blood transfusion and urine output. Hemoglobin (Hb) was checked every each 8 hours and blood transfusion was done if Hb was lower than 8 g/dl or the patient was in the state of shock. Rebleeding was suspected if persistent tarry stool, reappearance of hematemesis, orthostatic hypotension, unstable vital sign (blood pressure≤90 mmHg, heart rate≥120 per/min) or Hb drop≥2 g/dl, (despite blood transfusion) developed after the first endoscopic therapy. Patients suspected to rebleeding were evaluated by urgent endoscopy and if active bleeding, fresh blood or blood clots were seen, rebleeding was documented. In such cases, endoscopic therapy with epinephrine injection and electrocoagulation (by Argon plasma coagulation) was done to stop bleeding.

### 2.3 Statistical analysis

Statistical analysis was performed using statistical analysis of SPSS software (Version 11.5, Chicago, USA). The descriptive variables such as mean, standard deviations and frequency were used. With the *χ*^2^-tests and independent *t*-tests, the analysis for continuous variables and categorical variables are implemented respectively. All data are described with proper features, such as percentage, average and median. *P* value less than 0.05 indicates that there is statistical significance.

## 3 Results

**Figure 1:**
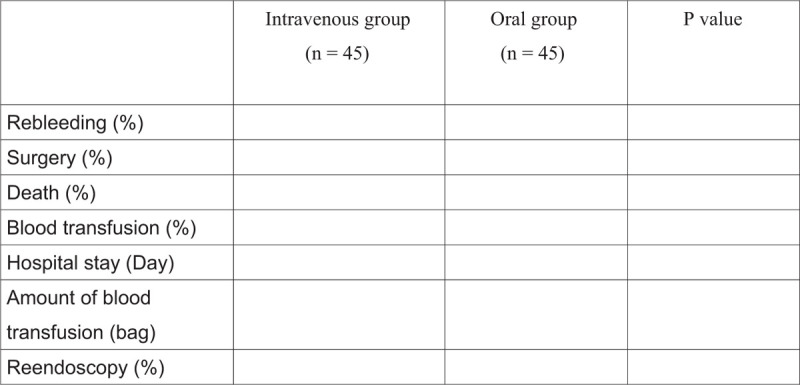
showed the primary and secondary end points.

Figure 1 showed the primary and secondary end points.

## 4 Discussion

Endoscopic therapy decreases but does not eliminates the risk of adverse outcome in peptic ulcer bleeding.^[15,16]^ On the other hand, gastric acid antagonizes hemostasis in the stomach and duodenum by impairing clot formation and promoting clot lysis.^[17]^ So, maintenance of intragastric pH>6 has been considered to result in a lower rebleeding rate of peptic ulcer. In recent years, several studies have shown the efficacy of intravenous PPIs in reducing the adverse outcome of peptic ulcer bleeding, despite the optimal dose, and the best route of administration has remained controversial.^[18]^ However, intravenous administration of PPIs has limitations. They are expensive, require a dedicated intravenous line, need nursing supervision and hospital admission. So, it would be reasonable to prescribe oral PPIs to patients with high risk bleeding ulcers provided that it is as effective as its intravenous counterpart. Oral PPIs have a high bioavailability. Its effect initiates one hour after ingestion and the maximal plasma concentration is achieved after 2–3 hours. However there are few studies comparing oral and intravenous PPI in decreasing risk of rebleeding in peptic ulcer patients.

Several limitations could be considered in our study. Firstly, we administered the drugs on admission and before the endoscopic therapy. Considering the fact that the presence of blood in stomach causes proton pumps activation and their subsequent irreversible deactivation by PPIs, we administered PPI on admission and before endoscopic intervention. Secondly, we did not calculate the Rockall score to determine if both groups have equal risk of rebleeding. Thirdly, different doses and types of PPIs were not analyzed. More high quality randomized controlled trials are still necessary.

## Author contributions

Lin Zhang planned the study design. Panya Diao reviewed the study protocol. Jing Zhang finished the manuscript. All of the authors approved the article and there is no conflict of interest.

**Conceptualization:** Panya Diao.

**Data curation:** Panya Diao.

**Funding acquisition:** Lin Zhang.

**Writing – original draft:** Jing Zhang.
